# Assessing high-impact spots of climate change: spatial yield simulations with Decision Support System for Agrotechnology Transfer (DSSAT) model

**DOI:** 10.1007/s11027-015-9696-2

**Published:** 2016-02-06

**Authors:** Anton Eitzinger, Peter Läderach, Beatriz Rodriguez, Myles Fisher, Stephen Beebe, Kai Sonder, Axel Schmidt

**Affiliations:** 1 CIAT International Center for Tropical Agriculture, A.A. 6713 Cali, Colombia; 2 CIAT International Center for Tropical Agriculture, Managua, Nicaragua; 3 CIMMYT International Maize and Wheat Improvement Center, Mexico DF, Mexico; 4 CRS Catholic Relief Services, Lima, Peru

**Keywords:** Climate change, DSSAT drybean submodel, High-impact spots, Simulation modeling, Central America

## Abstract

Drybeans (*Phaseolus vulgaris* L.) are an important subsistence crop in Central America. Future climate change may threaten drybean production and jeopardize smallholder farmers’ food security. We estimated yield changes in drybeans due to changing climate in these countries using downscaled data from global circulation models (GCMs) in El Salvador, Guatemala, Honduras, and Nicaragua. We generated daily weather data, which we used in the Decision Support System for Agrotechnology Transfer (DSSAT) drybean submodel. We compared different cultivars, soils, and fertilizer options in three planting seasons. We analyzed the simulated yields to spatially classify high-impact spots of climate change across the four countries. The results show a corridor of reduced yields from Lake Nicaragua to central Honduras (10–38 % decrease). Yields increased in the Guatemalan highlands, towards the Atlantic coast, and in southern Nicaragua (10–41 % increase). Some farmers will be able to adapt to climate change, but others will have to change crops, which will require external support. Research institutions will need to devise technologies that allow farmers to adapt and provide policy makers with feasible strategies to implement them.

**Electronic supplementary material:**

The online version of this article (doi:10.1007/s11027-015-9696-2) contains supplementary material, which is available to authorized users.

## Introduction

Over the past decades, assessments of climate change impacts on agricultural crop production using empirical and process-based modeling have emerged for generating useful information and recommendations of adaptation strategies (Challinor et al. 2009). Several studies show the potential impacts of climate change on agriculture that may add significant challenges of ensuring food security and reaching global development goals (Morton 2007; Jarvis et al. 2011b; Teixeira et al. 2013; Rosenzweig et al. 2014). Jones and Thornton (2003) state a decrease in yield for maize by 2055 in Africa and Latin America due to progressive climate change will likely only be 10 %, but it represents equivalent losses of US$2 billion per year.

Smallholders will suffer most from climate change, and impacts will be locally specific and difficult to predict without remaining highly uncertain (Jarvis et al. 2010). To identify the geographical regions and spatial patterns of crop exposure to climate change is a crucial step in risk assessment and the development of the right adaptation strategies (Turco et al. 2015). Mechanistic models are widely accepted in agricultural research to understand crop-climate suitability and productivity (Ramirez-Villegas et al. 2011; Estes et al. 2013). Crop simulation models can also identify the potential impact of long-term changes of climate patterns on crop suitability and production (Jones and Thornton 2003; Beebe et al. 2011; Laderach et al. 2011; Jarvis et al. 2011b; Cortés et al. 2013). Keating and McCown (2001) reviewed biophysical simulation models that have evolved over the last 40 years. They recognized the strength of the generic grain cereal simulation model CERES and the CROPGRO model for grain legumes (Hoogenboom et al. 1994) to simulate crop yield responses to management factors. They also recognized their weakness to deal with integrated cropping systems. In recent years, these limitations were overcome in integrated modeling frameworks like the Agricultural Production Systems Simulator (APSIM) (McCown et al. 1996) and Decision Support System for Agrotechnology Transfer (DSSAT) (Jones et al. 2003). Simulation modeling has been used to highlight the impact of climate change on crop production and the vulnerability of farming communities (Jarvis et al. 2011a; Bellon et al. 2011; Baca et al. 2014; Eitzinger et al. 2014). Some of these studies used modeling to develop possible strategies to adapt to climate change in the region (Lobell et al. 2008; Ravera et al. 2011; Jarvis et al. 2011a; Ramirez-Villegas et al. 2012).

Drybeans (*Phaseolus vulgaris* L.) and maize (*Zea mays* L.) were the main food staples of the pre-Columbian civilizations in Central America. Drybeans remain part of the culture (Leterme and Muñoz 2002) and are an important subsistence crop in Central America. Consumption is higher than elsewhere in Latin America (FAO 2009) (Table 1), with Nicaragua’s per capita consumption ranking third globally (FAO 2009) (Table 2). In El Salvador, Guatemala, Honduras, and Nicaragua, more than one million smallholder families depend on drybeans for their livelihood, with commercial production of 0.5 million tons per year (Instituto Interamericano de Cooperación para la Agricultura (IICA-Nicaragua) 2007). Consumption has changed little over the last 35 years in rural communities of Central America (Leterme and Muñoz 2002), reflecting tradition and their geographical isolation. Rural families depend on drybeans produced locally, which are not traded commercially.

**Table 1:** Consumption of drybeans in Latin America (FAO 2009)

Country	Drybean consumption
	kg/capita/year
Nicaragua	23.4
Brazil	16.3
El Salvador	15.2
México	10.3
Costa Rica	9.3
Honduras	8.7
Guatemala	8.3
Belice	6.4
Paraguay	5.8
Venezuela	5.3
Colombia	3.5
Perú	2.1
Panamá	1.8
Chile	1.6
Uruguay	1.1
Ecuador	0.4
Argentina	0.3
Suriname	0.3
Bolivia	0.2
Guyana	0.1

**Table 2:** World consumption of drybeans (FAO 2009)

Country	Drybean consumption
	kg/capita/year
Rwanda	29.3
Burundi	26.0
Nicaragua	23.4
Cuba	16.6
Brazil	16.3
El Salvador	15.2
Tanzania	14.2
Benin	13.7
Korea	12.5
Cameroon	11.6
Kenya	11.1
Uganda	10.9
Togo	10.5
Mexico	10.3
Costa Rica	9.3
Honduras	8.7
Guatemala	8.3
Haiti	7.9
Angola	7.5
Timor-Leste	7.3

Unlike the gene pool of Andean drybeans, which is adapted to cooler climates, the Mesoamerican gene pool is adapted to higher temperatures at low to medium altitudes (400–2000 m above sea level (masl)) (Beebe et al. 2011). Nevertheless, environments suitable for growing drybeans in Central America are most limited by maximum temperatures (Beebe et al. 2011). This limitation is likely to become more important as temperature increases through global warming.

We developed a method to identify spatial patterns as hotspots by quantifying statistical outliers of predicted changes in future yields and tested the method on a pilot study in Central America, covering an area of four countries: El Salvador, Guatemala, Honduras, and Nicaragua. We used the drybean sub model of DSSAT to assess the impact climate change will have on drybeans. We show results of change in productivity for main drybean-producing regions, and derive conclusions for drybean breeding and adaptation.

Central America has five Köppen climate zones (Peel et al. 2007): tropical rainforest (Af), tropical monsoon (Am), tropical savanna (Aw), humid subtropical (Cwa), and dry (arid and semiarid) (Bw) climates. In Central America, drybeans are produced in tropical savanna climates, which have distinct wet and dry seasons of equal duration. In suitable areas, the wet season extends May–October, followed by a marked dry season. Annual rainfall is influenced by topography, with inter-annual variability as much as 750 mm (Ravera et al. 2011).

Precipitation in Central America is distributed bimodally, with less rainfall and higher temperatures during the dry spell in July and August between the two rainy seasons (called *canícula* in Spanish; Magaña et al. 1999). The canicula separates the two main growing seasons on the Pacific side of the isthmus where most of the population lives and where there is the most agriculture. The *primera* season May–mid July is followed by the *postrera*, September–November after the canicula. There is a third growing season, the *apante* (December–February), on the Atlantic side with Am climates (Costa Rica, south and southeast Nicaragua, eastern Honduras, and northern Guatemala). Planting in the *apante* has increased in the last two decades in response to the demand caused by the long dry season on the Pacific side.

Central America has warmed over the last decades (Aguilar et al. 2005), with more extreme high temperatures that are spatially highly coherent. Rainfall increased somewhat over the last 40 years on most of the Caribbean side of Central America and southern Mexico. The canicula on the Pacific coast became more pronounced (Aguilar et al. 2005).

In view of the importance of drybeans for Central America, this study was conducted to assess spatial high-impact spots of climate change on crop production and provide general recommendations for priorities if strategies should focus on diversification, intensification of existing production systems, or conservation.

## Methods and data

To analyze and understand potential impacts of climate change on crop production on a regional scale, we applied the following steps:

- We selected a range of model treatments that represent farming practices of drybeans in the target countries.
- We identified high-impact spots where climate change will impact drybean production in the first planting season of the year (in Central America called *primera*).
- We compared simulated impacts on drybean yields for the second (called *postrera*) and the third (called *apante*) seasons on selected sites within the different types of hotspots.
- We used data from multiple global circulation models (GCMs) for selected sites to assess uncertainty.

**Fig. 1:**
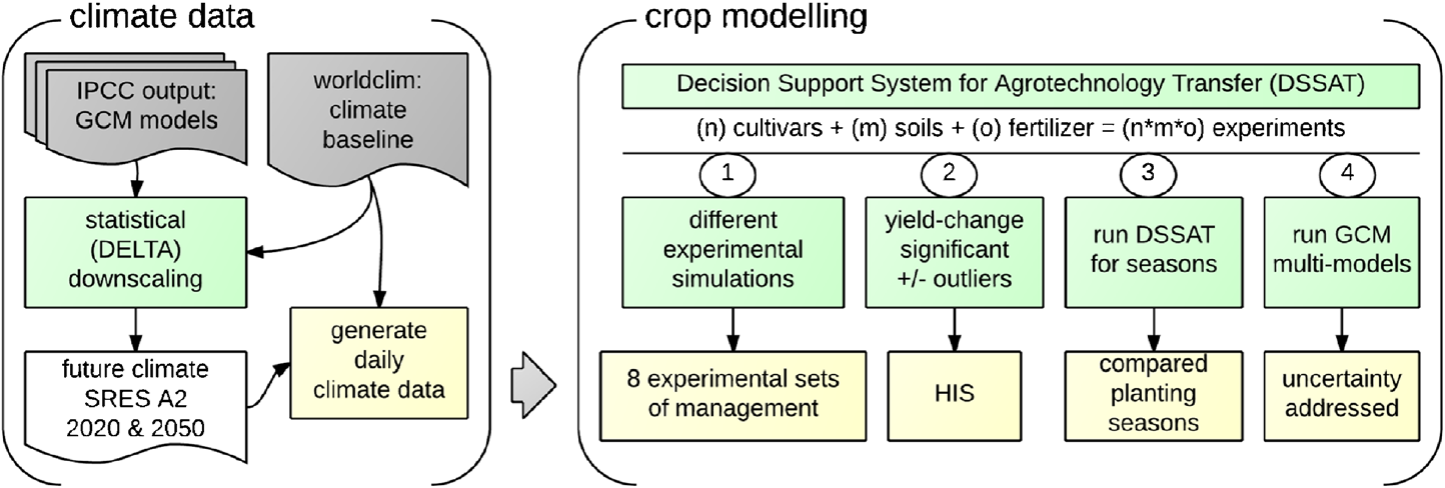
The sequence followed in the simulation modeling

We used WorldClim (Hijmans et al. 2005) and downscaled GCMs (Ramirez-Villegas and Jarvis 2010) to provide monthly climate data for the climate baseline (current climate) and future climates. We generated daily climate from the monthly data, which we then used in the DSSAT drybean model (Fig. 1). We describe each procedure in more detail below.

### Climate data

We used monthly total precipitation and mean monthly minimum and maximum temperature data as input to the MarkSim weather generator. For the climate baseline, we used the WorldClim database (Hijmans et al. 2005), which interpolated between observed data from more than 47,000 weather stations worldwide for the period 1950–2000 (Hutchinson 1995).

For future climates, we used the GCMs that the Intergovernmental Panel on Climate Change (IPCC) used for its “Fourth Assessment Report (AR4)” (Intergovernmental Panel on Climate Change (IPCC) 2007). We selected the GCMs’ outputs for the A2 scenario from the IPCC’s Special Report on Emissions Scenarios (SRES) (Intergovernmental Panel on Climate Change (IPCC) 2000). The A2 scenario describes a very heterogeneous world with high population growth, slow economic development, and slow technological change. It is commonly called the “business as usual scenario,” and 13 years after publication of the SRES, it reflects the current situation.

The spatial resolution of the GCMs (1–2°) is inappropriate for analyzing the impacts on agriculture (Jarvis et al. 2010), which therefore needs downscaling to provide higher resolution surfaces. We used the delta method of downscaling (Ramirez-Villegas and Jarvis 2010), which is based on the sum of interpolated anomalies to 30″ monthly climate surfaces of WorldClim. The method assumes that changes in climates are only relevant at coarse scales and that relationships between variables will be maintained in the future.

We used downscaled data from all 19 GCMs from IPCC’s AR4 for two different 30-year running-mean periods, 2010–2039 [2020s] and 2040–2069 [2050s]. We took means of the 30″ data (Ramirez-Villegas and Jarvis 2010) to produce 2.5′ and 5′ spatial resolution (roughly 5 and 10 km) for Nicaragua, Honduras, El Salvador, and Guatemala. We used the monthly data for each 2.5′ pixel as input to the MarkSim climate generator to produce daily weather data (Jones and Thornton 1993, 2000; Jones et al. 2002).

MarkSim uses a third-order Markov function to generate daily weather data that reflects the synoptic control of rainfall in the tropics by convection cells. It generates daily data of maximum and minimum temperatures, rainfall, and solar radiation for as many years as the user requires. We generated 99 replicate years of daily weather data for the climate baseline and for each of the 19 GCM models for the 2020s and 2050s for each pixel in the four countries. We automated this step by using the MarkSim 1.0 code compiled as an executable and running it from a command line under the control of a master FORTRAN procedure. In this way, we were able to run the process unattended as the run of MarkSim for each site was independent. The master procedure logged any failures of MarkSim but continued with the next site, which was not possible using MarkSim’s shell routine in batch mode.

### Crop modeling

DSSAT is a widely tested series of simulation models (Jones et al. 2003; Hoogenboom et al. 2010). It incorporates detailed understanding of crop physiology, biochemistry, agronomy, and soil science to simulate performance of the main food crops, as well as pastures and fallows. It simulates crop water balance, photosynthesis, growth, and development on a daily time step. DSSAT requires input of the soil water characteristics and genetic coefficients of the crop cultivar, plus any relevant agronomic inputs such as fertilizer and irrigation. It is driven by daily maximum and minimum temperatures, rainfall, and solar radiation.

**Fig. 2:**
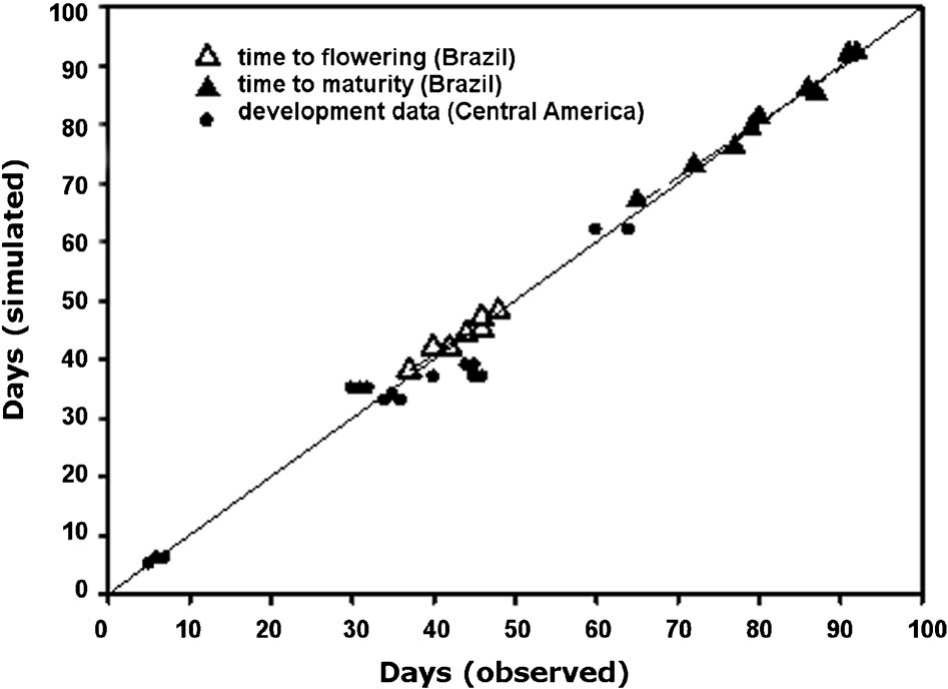
Simulated compared with observed phenological data for drybeans grown in southern Brazil (Oliveira et al. 2012) and in ancillary experiments at three sites in Central America

BEANGRO is a simulation model for drybeans (*P. vulgaris* L.) that was integrated into the crop simulation module component of DSSAT (Hoogenboom et al. 1994). It simulates vegetative growth, reproductive development, and yield. It has been validated many times (see, for example, Oliveira et al. 2012) and accurately reflects the phenological development and yield of drybean cultivars (see, for example, Oliveira et al. 2012 and Fig. 2). Here, we used it to examine the difference between yields using the climate data described above.

We prepared DSSAT management files (FILEX) that included initial conditions at planting, cultivar selection, planting data, and row and plant spacing, among others. We consulted experts from the CIAT bean program and from national bean programs in the four countries in Central America on the appropriate management to apply.

We assessed final impact using the mean of the treatments and calculated the anomalies across sites (pixels) of future and baseline yields.

### Modeling steps

#### Simulations of bean management for the *primera* season

We defined a sowing window between 15 April and 30 June (the *primera* planting season) with a sowing trigger of 50 % available soil water in the top 30-cm layer of soil. The simulations started with available soil water (ASW) set at the lower limit (−1.5 MPa water potential) 60 days before the start of the sowing window to allow early season rain to accumulate in the soil. In consultation with experts, we selected one cultivar (black-seeded ICTA OSTUA) and one breeding line (red-seeded BAT1289) representative of cultivars commonly used in Central America. Because we could not obtain spatially distributed soil data, we used representative generic medium sandy loam and medium silt loam soils from the DSSAT package. We simulated two levels of fertilizer applications, 64 kg/ha 12-30-06 and 128 kg/ha 18-46-00 (N-P-K) at sowing, both with a side dressing of 30 kg N/ha as urea 22 days after sowing. The design was therefore a factorial arrangement of two cultivars, two soils, and two levels of fertilizer:

$$ \left\{\begin{array}{c}\hfill \mathrm{ICTA}\ \mathrm{OSTUA}\hfill \\ {}\hfill \mathrm{BAT}1289\hfill \end{array}\right\}\times \left\{\begin{array}{c}\hfill \mathrm{generic}\ \mathrm{medium}\ \mathrm{silty}\ \mathrm{loam}\hfill \\ {}\hfill \mathrm{generic}\ \mathrm{medium}\ \mathrm{sandy}\ \mathrm{loam}\hfill \end{array}\right\}\times \left\{\begin{array}{c}\hfill 64\;\mathrm{kg}/\mathrm{ha}\ 12\hbox{-} 30\hbox{-} 06(F1)\hfill \\ {}\hfill 128\;\mathrm{kg}/\mathrm{ha}\ 18\hbox{-} 46\hbox{-} 00(F2)\hfill \end{array}\right\} $$

Equation 1: experimental design used in DSSAT

The lower level of fertilizer represents a typical farmer’s management in Central America. A more advanced farmer might use the higher level, which also gives an estimate of the potential yields of the selected cultivars.

We used the averaged climate for the 19 GCMs as input data in a first step at 5′ resolution. After identifying high-impact spots (see below), we ran the simulations at 2.5′ using all 19 GCMs in step 4 (see Section 2.3.4).

#### Identify future high-impact spots

We calculated the yield change (future-baseline) from the yield outputs of the simulations (the mean of the eight treatments in step 1). We used the climate baseline and the ensemble of future climate data from the GCMs. We used distance statistics (Getis and Ord 1992) to identify the significant outliers and the high-impact spots (HISs).

Distance statistics analyze spatial association by measuring the degree of association within a population of weighted points. Spatial association is when the deviation of the variable of interest with respect to the mean (*z*-value) is greater than some specified level of significance. Here, we used a robust version of the root mean square (Darrouzet-Nardi and Bowman 2011) to scale the data and identify points that lie outside positive and negative cutoffs.

We used the HISs to identify priorities for diversification, adaptation, or conservation strategies. The three types of HISs were:

- Adaptation spots: We identified pixels whose negative *z*-values of spatial association were equal to or greater than one standard deviation of the mean (68 %). We expect that yields of drybean in the *primera* season in these pixels will decrease in the 2020s and even more in the 2050s.
- Hotspots: Pixels whose negative *z*-values were greater than two standard deviations of the mean (95 %). Yields will be so low that it will probably not be economic for farmers to continue to grow drybeans.
- Pressure spots: Pixels whose positive *z*-values are greater than one standard deviation of the mean are where the future climate will favor drybeans. Most of these pixels lie outside the current zone of bean production, but we did map them (see Section 3).

We identified hotspots and adaptation spots within the areas that currently grow drybeans. We overlaid the pixels on maps from the Bean Atlas for the Americas (Mejia et al. 2001) using a kernel density analysis (Silverman 1986). By this means, we also identified the pressure spots outside the areas that currently grow drybeans.

#### Comparison of different growing seasons for selected sites and estimation of fertilizer responses

Changing planting dates would be an adaptation option, if alternate growing seasons were to give a yield advantage in future climates. We therefore ran the same set of treatments for the *postrera* and *apante* planting seasons and compared results with those for the *primera*. Then, within the identified hot- and adaptation spots, we selected 15 communities within municipalities that produce drybeans, distributed across all four countries. We selected pixels that lay within 15 km of the selected communities that intersected with the bean-growing areas identified in the Bean Atlas. We constrained the selection to those pixels whose elevation lay within 100 m of the elevation of the selected community. In total, we selected 171 points for the comparison between seasons (Table 3).

**Table 3:** Selected municipalities and points used from the Bean Atlas for simulating different planting seasons

Country	Municipality	Points	Elevation range (masl)	Country	Municipality	Points	Elevation range (masl)
El Salvador	Apastepeque (AS)	30	278–375	Honduras	Alauca (HS)	3	567–639
	Candelaria (AS)	11	524–575		Danli (AS)	12	716–798
	Comasagua (AS)	14	482–574		Orica (HS)	10	811–900
	Texistepeque (AS)	28	603–702		Yorito (HS)	5	743–791
Guatemala	Ipala (AS)	14	793–857	Nicaragua	La Conquista (HS)	11	229–320
	Jalapa (AS)	5	1491–1556		San Dionisio (AS)	9	305–393
	Parramos	4	1755–1801		Totogalpa (HS)	11	664–696
	Patzicia	4	2104–2185				

*HS* hotspots, *AS* adaptation spots

We defined the planting date windows 15 April–30 June for the *primera* season, 20 August–30 September for the *postrera*, and 25 October–5 December for the *apante*. We also ran the model without simulating nutrient options to assess the fertilizer response on each site. We estimated the yield with no fertilizer increase by disabling the fertilizer application in the simulation control options. We did this for the 15 selected sites using current and climate input data for climate baseline and GCM ensembles for the 2020s and 2050s.

#### Run data from multiple GCMs on selected sites for the *primera* season to assess the prediction uncertainty

Uncertainty in climate projections raises doubts as to their applicability in crop models (Asseng 2013). Acknowledging that uncertainty exists is the first step towards being able to quantify it (Challinor et al. 2009; Ramirez-Villegas et al. 2013). We used data from all 19 GCMs on the 171 points selected in the previous step to generate daily data for the 2020s and calculated the change of yield for each GCM. For each point, we estimated the uncertainty of the simulated yields for the predicted future climates:

- The yield change of the GCM ensemble mean
- The standard deviation (SD) of the yield change
- The agreement among the model simulations using the 19 GCMs’ climate projections, calculated as the percentage of the model outputs predicting changes in the same direction

## Results

We present the data as maps for El Salvador, Guatemala, Honduras, and Nicaragua.

### Impact on yields for the first season *primera*

**Fig. 3:**
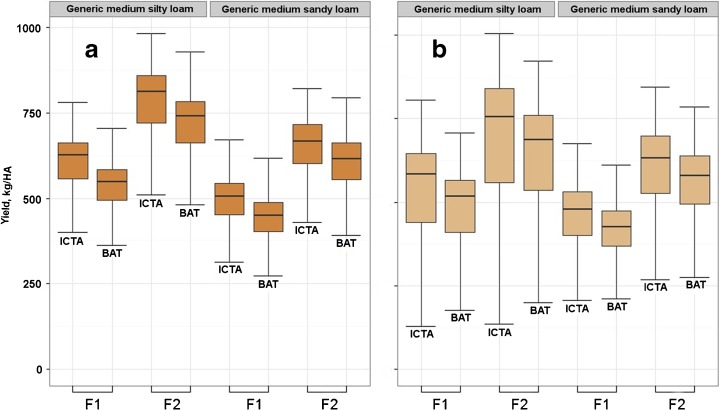
Yields over 4439 points in four Central American countries of the drybean cultivars ICTA Ostua (ICTA) and BAT1289 (BAT) at two levels of fertilizer (*F1*, *F2*) and two soils (Generic medium silty loam, Generic medium sandy loam) with **a** baseline climate and **b** 2020s future (using the GCM ensemble)

Figure 3 shows simulated yields averaged over all sites in the four countries, comparing the current climate with the ensemble of GCMs for the 2020s. Mean yields decrease slightly but become a lot more variable.

**Fig. 4:**
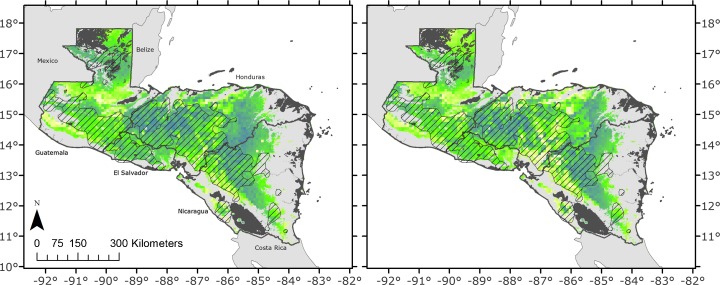
Mean simulated yield of eight treatments in the *primera* season in **a** baseline conditions and **b** 2020s future (using the GCM ensemble). Areas are colored from blue (high yields) to *yellow* (low yields)

In Nicaragua, the most decrease in yield in the 2020s will be in the departments of Granada (−38 %) and Carazo (−25 %). The greatest impact in tons produced is predicted for Nueva Segovia, Estelí, and Madriz. Constant or even improved yields are predicted for the eastern slopes of the central highlands, Jinotega and Matagalpa, which are the main bean-growing areas in Nicaragua (Fig. 4, Online Resource 1).

The corridor of yield decrease continues in Honduras through El Paraiso (−12 %), Francisco Morazan (−13 %), and Yoro (−10 %) departments. In southwest Honduras close to the El Salvador border, we expect high impact for the 2020s in Choluteca (−32 %), Cortes (−17 %), and Valle (−20 %) departments. We expect increased yields only in Ocotepeque department (Fig. 4, Online Resource 1).

In El Salvador, the simulations show reduced yields in the southeastern departments of Cuscatlan (−12 %), Cabañas (−10 %), and San Vicente (−14 %) in the 2020s. We expect yields to increase only Ahuachapan department (Fig. 4, Online Resource 1).

In Guatemala, drybean production in the 2020s will increase in San Marcos (+15 %) and Totonicapán (+16 %) departments. In contrast, Peten (−10 %) department, where there is now enough rainfall to support opportunistic *apante* sowings, will suffer the highest yield decrease for the *primera* sowings (Fig. 4, Online Resource 1).

### Identified high-impact spots

**Fig. 5:**
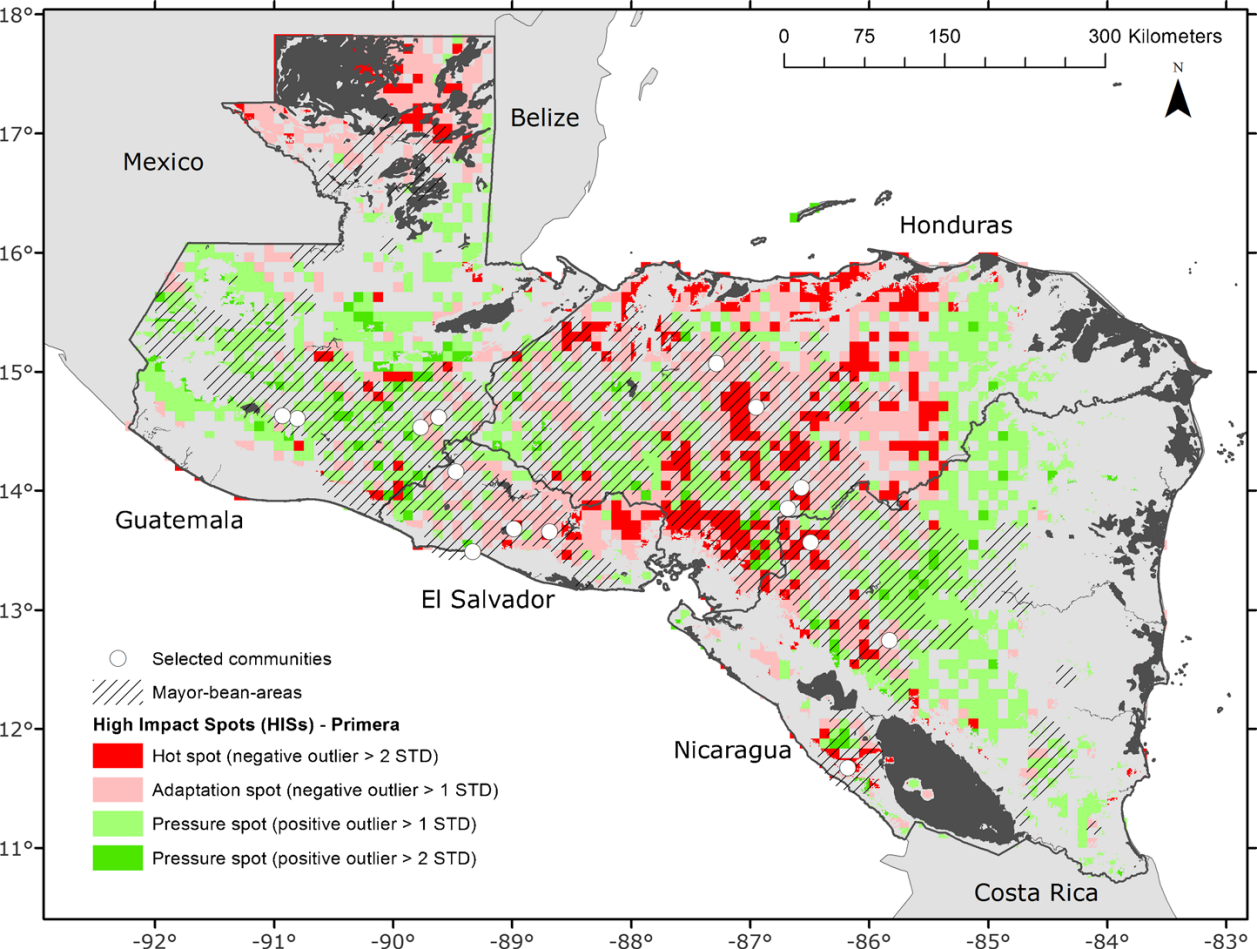
Outliers from yield change of drybeans in Central America in the *primera* planting season. Pressure spots are more than one standard deviation of the mean higher yield HIS (*green*), and hotspots are more than two standard deviations of the mean lower yield (*red*). Hatched areas are the main bean-growing areas; *white points* are the 15 selected bean production sites

We mapped the different categories of HISs so that we could suggest likely interventions at the farmer and national levels. The adaptation spots and the hotspots all lie within the areas that currently grow drybeans (hatched areas in Fig. 5, taken from the Bean Atlas), while the pressure spots generally lie outside them.

Negative HISs are concentrated from Lake Nicaragua to the northern coast of Honduras along the Central American dry corridor. Other areas currently used for drybean production and identified as positive HISs (green within the hatched areas) seem to be promising for future development of drybean production in the region (Fig. 5, Online Resource 2).

**Fig. 6:**
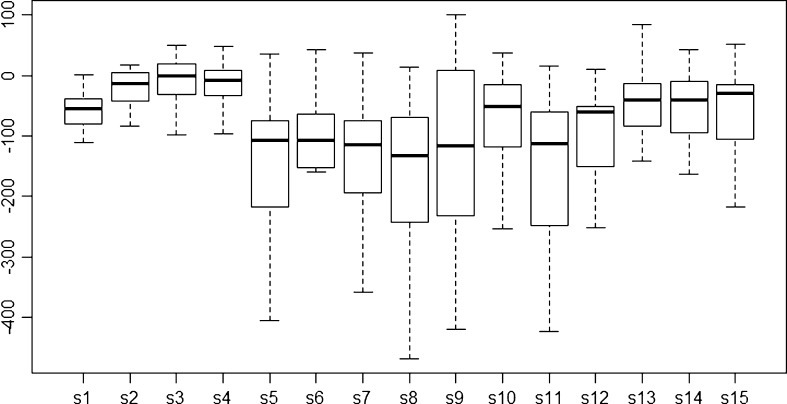
Yield change for 15 sites using 19 GCMs; Ipala (*s1*), San Dionisio (*s10*), Apastepeque (*s12*), Candelaria (*s13*), Comasagua (*s14*), and Texistepeque (*s15*) are adaptation spots (small negative yield change); Alauca (*s5*), Danli (*s6*), Orica (*s7*), Yorito (*s8*), La Conquista (*s9*), and Totogalpa (*s11*) are hotspots (large negative yield change). Yields at Jalapa (*s2*), Parramos (*s3*), and Patzicia (*s4*) are not expected to change

Further analysis of the detailed data shows yield changes for the 2020s using all 19 GCMs for all 15 sites (Fig. 6). Using the HIS analysis, we identified Alauca (s5), Orica (s7), Yorito (s8), La Conquista (s9), and Totogalpa (s11) as hotspots. At these sites, production of drybeans will probably not be possible in the future and farmers will need a strategy to diversify their crops.

Ipala (s1), Jalapa (s2), Danli (s6), San Dionisio (s10), Apastepeque (s12), Candelaria (s13), Comasagua (s14), and Texistepeque (s15) are adaptation spots in the main production areas. In these areas, drybean systems can be adapted if suitable measures are taken in the near future. Results are based on simulations only for the *primera* season. We selected adaptation and hotspots only within the current main production areas. Areas outside these are not used or not important for drybean production in the main seasons, although some of them are important for the *apante* season. We did not include sites of pressure spots, although Parramos (s3) and Patzicia (s4) in Guatemala showed small gains in productivity in the future scenarios.

### Comparison of alternative planting seasons

Simulations of different planting seasons for Guatemala show little change by the 2020s, even slight increases except for the *apante* season (Table 4). But El Salvador and, more severely, Honduras can expect up to 9 % yield loss in the *primera* planting season in municipalities that currently produce half the countries’ commercial beans. The *postrera* season shows little loss in all countries, and even some gains. In El Salvador and Guatemala in the *apante* season, 25 and 20 % of municipalities respectively will have losses greater than 10 %.

**Table 4:** Yield loss for the first season *primera*, second season *postrera*, and third season *apante* by country predicted for the 2020s

Country	Average change in bean yield [%]		% of municipalities with expected yield loss >10 %
	All municipalities	In municipalities accounting for 50 % of total production	
	*Primera*/*postrera*/*apante*	*Primera*/*postrera*/*apante*	*Primera*/*postrera*/*apante*
El Salvador	−7/−1/−7	−6/−1/−10	33/0/25
Guatemala	+1/+6/−2	−2/+5/−8	10/0/20
Honduras	−9/0/−4	−9/−1/−5	43/0/14
Nicaragua	−8/+7/−4	0/+2/−3	29/0/12

**Fig. 7:**
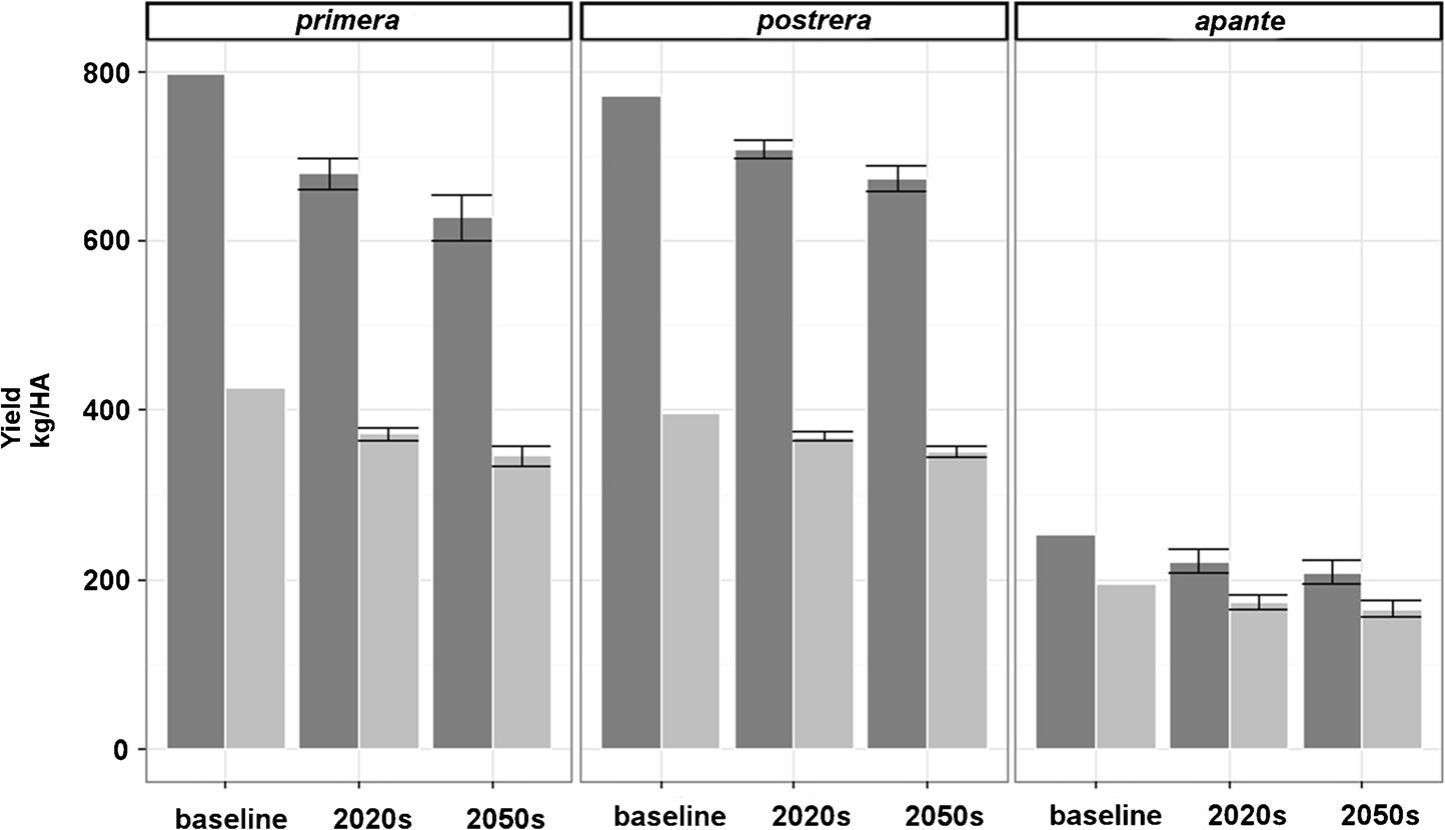
Mean yields of drybeans at 15 sites (171 points) for three planting seasons for baseline and future climates; *dark grey bars* show yields with higher fertilizer (F2); *light grey bars* show yields with nutrients not simulated. Standard deviations are for all 19 GCMs for 2020s and 2050s

Yields for the 171 points within the 15 selected sites show that the *primera* season is likely to be more affected (16 % less by the 2020s and 23 % less by the 2050s) than either the *postrera* or *apante* season (Fig. 7). The *postrera* may therefore become more important for farmers than the *primera* although the *postrera* yields will also decrease by 6 % in the 2020s and 16 % in the 2050s. The *apante* season, with yields of only 200 kg/ha, is only cropped opportunistically and will change little. The yields over the 15 sites were somewhat lower than those for the whole region (800–1000 kg/ha).

Fertilizer gives large increases in yield, and with no fertilizer, yields were only 34 % of those with 128 kg/ha 18-46-0. The yield response to fertilizer might be diminished by less favorable climates in the future. Fertilizer has only a small effect in the low-yielding *apante* season.

### Uncertainty of the GCM results

**Fig. 8:**
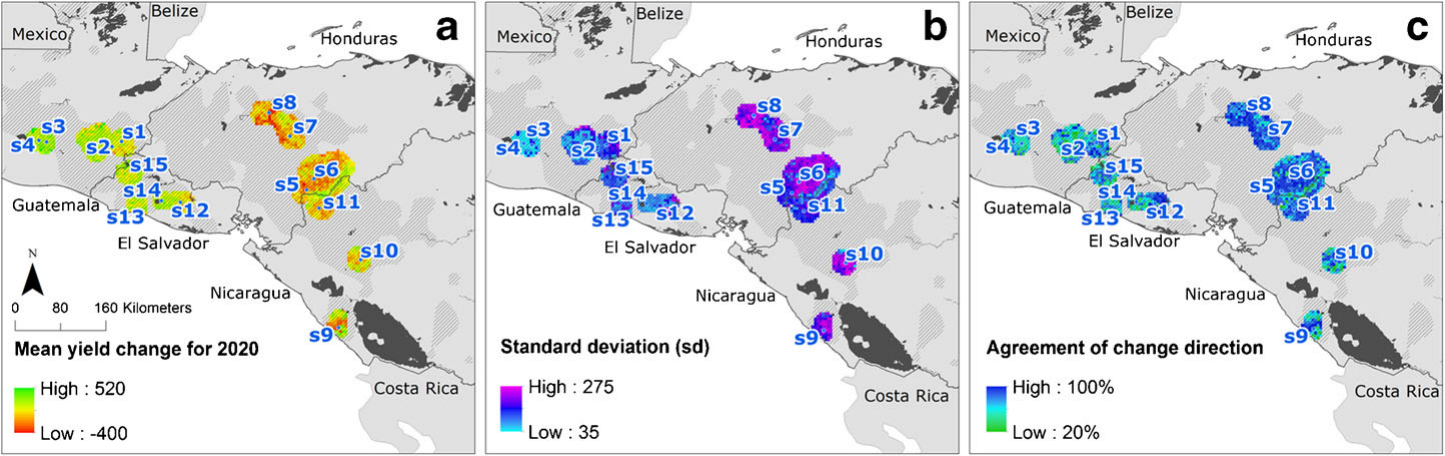
Predicted changes in yields of drybeans and the range of uncertainty of the GCM outputs: **a** mean yield change for 2020s for the ensemble of 19 GCMs, **b** SD of the mean of yield change for the 19 GCMs, and **c** percentage of GCMs agreeing in the direction of simulated yield change. *Hatched areas* are main drybean-growing areas. The *subfigures* correspond to the categories described in Section 2.3.4

Here, we consider the skill of the GCMs to forecast climate as it affects bean yields in the *primera* growing season. To do so, we used the predictions of each GCM separately as input to the DSSAT drybean submodel for all 171 points within the 15 selected sites. Simulated yields using the 19 different GCMs varied widely (Fig. 8). The change in the simulated yields using the 19 different GCMs varied widely across sites (Fig. 8a), but in general, the GCMs agreed in the direction of yield change (Fig. 8c). The SDs of the means of change in yields for the future climates for each site are a measure of the confidence of the predictions. Where the SDs are high, the uncertainty of prediction of the variability is higher (Fig. 8b) as for sites identified in light blue colors in Guatemala and El Salvador. In contrast, in Honduras and Nicaragua, where it is only locally lower, the sites with lower uncertainty should confront climate change with more certainty, although they must be combined with data of yield changes, which indicate whether yields in the future will be better or worse.

We can discern some geographical separation. The more mountainous sites (s5–s11) appear to have greater uncertainty in the prediction of yield change. We must caution that this may not be just the GCMs themselves, but the uncertainties introduced by the downscaling, by WorldClim and by MarkSim.

## Discussion

Global food systems require locally specific urgent action to reduce vulnerability of a highly sensitive agriculture in the face of climate change (Vermeulen et al. 2011). Hotspot mapping can help to identify regions that are particularly vulnerable to future climate impacts, with the goal of drawing policy-maker attention to target adaptation measures (de Sherbinin 2014).

For our study area Central America, the general analysis identified adaptation spots and hotspots where climate change will cause modest and severe reductions in yield. In pressure spots, there will be modest yield increases. The more detailed analysis showed differences between planting seasons and the uncertainties between the GCMs. These analyses met our overall goal to differentiate areas that will require different measures for farmers and policy makers to cope with climate change.

Farmers in adaptation spots will have to adjust their management if they are to continue growing drybeans in the future, for example, by sowing better-adapted cultivars. In hotspot pixels, farmers will need to diversify their livelihoods because it will likely be uneconomic to grow drybeans. Future strategies might be to diversify to other crops, seek off-farm income, or leave agriculture.

Pressure spots mostly lie outside the current bean-growing zone. They are often located in forest reserves or at higher altitudes, or are close to the current agricultural frontier. There will be social and political pressure to allow agriculture to migrate into these areas. Identifying pressure spots is important so that national and regional decision-makers can either develop them in an ecologically sustainable way or protect them.

The more specific analysis differentiated planting seasons and the potential of fertilizer to increase yields in the selected municipalities. In the future, the *postrera* planting season will become more important in these municipalities. Fertilizer still increased yields with climate change, although the cultivars ICTA OSTUA and BAT1289 that we used appeared to become somewhat less responsive to fertilizer. Beebe (Beebe et al. 2014) argues that edaphic factors will become important as climate change will bring more frequent droughts. A more comprehensive study using site-specific soil data and data from field experiments on the effects of fertilizer and improved varieties is necessary to verify this argument.

We focused first on likely effects of climate change on drybean production in the *primera* planting season. We then assessed the potential for the *postrera* planting season at adaptation spots and hotspots defined in the *primera*. For many of these sites, the *postrera* planting season will be more favorable. We included the *apante* season, which is largely on the Atlantic coast where droughts are rare but production is opportunistic and yields are low. The *apante* crop grows in a period of falling temperatures so that climate change may make the crop more attractive. Bean production has expanded on the Atlantic coast and will likely continue as production in the *primera* season becomes less favorable elsewhere. Further studies of climate change should include this region to test this hypothesis.

There is a great potential to improve insights on future production constraints using multiple GCMs and a wider range of scenarios for spatially distributed DSSAT simulations. Using a dataset containing historical daily weather data and daily future predictions would be another refinement of the methodology we present here. In areas where detailed soil data are available, they should replace the generic soils we used in our simulations.

We also need physiological and phenotypic data on the growth and development in the field of regional and promising cultivars to determine their crop-specific coefficients for DSSAT. With these data, we could generate virtual varieties with heat and drought tolerance, which could help identify the potential of genetic improvement to adapt to climate change. It would also allow us to evaluate strategies of crop management oriented towards adapting current bean production to future climates.

We caution that the fertilizer utilization in the three different seasons needs to be investigated in more detail. The research should consider wider ranges of treatments and the effect of P, which is not implemented in the current DSSAT drybean submodel. Future work should also include the CO_2_ fertilization response, for which we need more experimental data on which to base the modeling.

GCMs do not predict future climates well for particular sites but rather estimate conditions on a large scale. GCM estimates can therefore not be used directly as input into plot-scale agriculture models (Ramirez-Villegas and Challinor 2012). Higher resolution climate models can improve results if (i) models are matched in scale, (ii) the skill of models is assessed and ways to create robust model ensembles are defined, (iii) uncertainty and model spread are quantified in a robust way, and (iv) decision-making in the context of uncertainty is fully understood (Ramirez-Villegas and Challinor 2012). It is therefore necessary to address uncertainty of the climate prediction models used. Methods of impact assessment are sensitive to uncertainties. We attempted to assess the inherent uncertainty by using 19 credible GCMs used by the IPCC in its AR4 (Jarvis et al. 2012). GCMs continue to improve their skill with regard to temperature, but unfortunately, their skill with regard to precipitation is progressing more slowly (Ramirez-Villegas et al. 2013).

Based on the results of this study, we make the following general recommendations to address future climate change in Central America:

1. Breed drybeans for improved adaptation to heat and drought stress (Beebe et al. 2011, 2013).
2. If economically viable, extend production into the dry season with lower temperatures using irrigation and water-harvesting systems combined with improved soil fertility management (Fox et al. 2005).
3. Start building farmers’ awareness of adaptation to climate change and stimulate adaptive behavior in a social-learning process (Grothmann and Patt 2005; Grothmann et al. 2013).

All the above assume that farmers will use optimal management of abiotic stress and biotic constraints. The development and implementation of adaptation strategies to face progressive climate change will depend also on the participation of all actors in the bean sector in Central America. Research institutions and policy makers will need to provide feasible strategies too.

## Electronic supplementary material

Below is the link to the electronic supplementary material.

Online Resource 1 Tables of average DSSAT simulated yields for departments in each country. (PDF 320 kb)

Online Resource 2 Maps of drybeans impact hot spots HIS for three planting seasons in four Central American countries. (PDF 943 kb)
