# Supplementary material for: Assessing high-impact spots of climate change: spatial yield simulations with Decision Support System for Agrotechnology Transfer (DSSAT) model
Source: Mitig Adapt Strateg Glob Chang. 2016 Feb 6;22(5):743–60. doi: 10.1007/s11027-015-9696-2 (PMC6054003; doi:10.1007/s11027-015-9696-2)
Supplement: Supplementary file 1 — Tables of average DSSAT simulated yields for departments in each country. (PDF 320 kb) [file 11027_2015_9696_MOESM1_ESM.pdf]

Mitigation and Adaptation Strategies for Global Change:

Assessing high impact spots of climate change: spatial yield simulations with Decision Support System for Agrotechnology Transfer (DSSAT) model.

Anton Eitzinger, Peter Läderach, Beatriz Rodriguez, Myles Fisher, Stephen Beebe, Kai Sonder, Axel Schmidt

Anton Eitzinger, CIAT International Center for Tropical Agriculture, Cali, Colombia; a.eitzinger@cgiar.org

---

Online Resource 1:

We present results of average DSSAT simulated yields for departments in each country.

The tables show current yield (CUR), standard deviation within the department (STD); yield by 2020s (2020), change of yield between current and 2020s in percentage (Chg); departments with blue shaded area-field are departments covering 50% of harvested area; red shaded Chg-fields are with decrease more than 10%; green shaded Chg-fields are with increase more than 10%.

Nicaragua		DSSAT simulated yield (kg/ha)														
	Area	Primera (1st season)				Postrera (2nd season)				Apante (3rd season)				Primera	Postrera	Apante
	(ha)	CUR	STD	2020	STD	CUR	STD	2020	STD	CUR	STD	2020	STD	Chg (%)	Chg (%)	Chg (%)
Matagalpa	46,818	727	97	723	140	705	64	723	74	661	229	649	222	0	2	-2
Jinotega	30,748	804	69	811	59	762	79	797	36	760	148	758	133	1	5	0
Atlántico Norte	30,702	688	69	691	74	673	61	674	71	754	78	728	86	1	0	-4
Atlántico Sur	30,435	595	92	586	108	616	59	613	62	711	135	670	131	-1	-1	-6
Nueva Segovia	22,696	797	86	728	180	807	51	787	104	558	204	556	219	-9	-2	0
Rio San Juan	11,335	656	133	656	130	630	96	625	92	594	216	580	217	0	-1	-2
Estelí	9,413	664	96	589	139	672	67	675	77	304	138	316	142	-11	0	4
Leon	8,051	469	138	430	132	495	138	532	142	174	47	166	47	-8	7	-4
Madriz	7,973	723	99	630	171	715	98	714	90	387	197	366	212	-13	0	-5
Chontales	3,980	667	169	627	229	667	66	702	64	625	228	590	247	-6	5	-6
Boaco	3,815	696	138	693	188	684	82	747	62	550	250	541	253	0	9	-2
Rivas	3,569	547	173	529	206	448	249	629	178	405	230	410	224	-3	41	1
Carazo	2,451	629	125	475	247	521	179	533	237	238	71	193	86	-25	2	-19
Chinandega	2,394	520	159	446	163	580	142	619	113	168	51	172	95	-14	7	3
Managua	2,323	481	115	454	118	547	92	624	114	181	43	173	52	-6	14	-4
Granada	1,577	581	110	358	210	520	160	638	89	248	61	188	51	-38	23	-24
Masaya	882	582	128	571	173	584	57	644	100	257	115	252	113	-2	10	-2
Total	219,162												average	-8	7	-4
													50% area	0	2	-3
													> 10% loss	29	0	12

Honduras

DSSAT simulated yield (kg/ha)

Honduras	Area (ha)	Primera (1st season) CUR	STD	2020	STD	Postrera (2nd season) CUR	STD	2020	STD	Apante (3rd season) CUR	STD	2020	STD	Primera Chg (%)	Postrera Chg (%)	Apante Chg (%)
F. Morazán	13,144	751	71	656	163	771	53	761	99	410	189	389	190	-13	-1	-5
Olancho	12,862	762	97	698	128	805	48	802	75	705	118	687	138	-8	0	-3
El Paraiso	11,127	725	127	639	180	778	76	750	106	477	245	467	248	-12	-4	-2
Comayagua	7,074	827	61	792	105	772	54	788	74	576	215	516	226	-4	2	-10
Copan	6,119	827	35	798	62	802	31	801	28	557	131	552	123	-3	0	-1
Yoro	5,679	697	146	626	187	791	54	793	68	727	128	679	131	-10	0	-7
Santa Bárbara	5,656	781	88	723	145	755	81	752	71	714	122	673	116	-7	0	-6
Lempira	5,586	805	68	789	75	715	90	735	71	342	118	330	112	-2	3	-4
Intibucá	4,607	796	39	791	69	688	54	710	55	339	145	346	144	-1	3	2
Choluteca	4,241	528	210	361	265	560	167	526	203	181	105	183	128	-32	-6	1
La Paz	2,291	755	83	722	175	672	66	664	143	248	82	245	111	-4	-1	-1
Cortes	2,101	705	121	585	195	771	75	743	74	783	85	768	85	-17	-4	-2
Ocotepeque	957	771	103	798	70	695	141	736	96	328	112	337	97	4	6	3
Valle	441	606	69	488	53	593	52	607	40	154	41	116	25	-20	2	-25
Total	81,885												average	-9	0	-4
													50% area	-9	-1	-5
													> 10% loss	43	0	14

El Salvador

DSSAT simulated yield (kg/ha)

El Salvador	Area (ha)	Primera (1st season) CUR	STD	2020	STD	Postrera (2nd season) CUR	STD	2020	STD	Apante (3rd season) CUR	STD	2020	STD	Primera Chg (%)	Postrera Chg (%)	Apante Chg (%)
Santa Ana	16,652	750	50	712	55	706	41	697	45	145	51	125	49	-5	-1	-14
La Libertad	13,294	743	46	714	39	660	53	656	47	153	37	142	28	-4	-1	-7
San Vicente	9,024	753	39	649	56	682	43	663	40	184	39	160	23	-14	-3	-13
Usulutan	8,959	685	179	662	31	622	0	636	37	179	17	170	14	-3	2	-5
San Salvador	7,428	752	43	691	27	683	39	680	26	152	44	139	18	-8	-1	-8
Cuscatlan	5,711	753	35	666	41	695	45	671	26	181	46	163	17	-12	-3	-10
Sonsonate	4,508	713	68	695	52	635	57	608	32	196	47	192	44	-3	-4	-2
Ahuachapan	4,471	683	92	693	75	606	77	608	59	178	57	173	41	2	0	-3
San Miguel	4,419	709	56	638	55	606	43	625	47	174	15	165	24	-10	3	-5
Cabañas	3,027	756	34	677	42	676	33	670	30	225	51	205	44	-10	-1	-9
Chalatenango	2,397	722	50	698	70	673	47	674	43	213	87	216	99	-3	0	1
Morazán	1,555	756	30	620	65	605	51	580	48	200	32	173	29	-18	-4	-13
Total	81,445												average	-7	-1	-7
													50% area	-6	-1	-10
													> 10% loss	33	0	25

Guatemala

DSSAT simulated yield (kg/ha)

Guatemala	Area (ha)	Primera (1st season) CUR	STD	2020	STD	Postrera (2nd season) CUR	STD	2020	STD	Apante (3rd season) CUR	STD	2020	STD	Primera Chg (%)	Postrera Chg (%)	Apante Chg (%)
Petén	35,383	730	70	655	101	703	54	687	71	747	79	723	90	-10	-2	-3
Jutiapa	28,222	666	128	634	143	623	108	629	127	150	68	136	65	-5	1	-9
Quiche	20,733	657	125	673	103	597	182	628	172	513	323	507	316	2	5	-1
Chiquimula	17,621	723	85	706	107	703	80	722	63	261	154	229	157	-2	3	-12
Huehuetenango	16,859	625	209	666	190	542	222	597	218	367	324	375	316	7	10	2
Jalapa	13,329	673	83	691	77	637	103	665	88	263	136	248	125	3	4	-6
Santa Rosa	12,571	649	72	629	89	593	47	605	73	195	75	164	71	-3	2	-16
Guatemala	9,511	677	25	696	45	651	32	672	41	157	47	136	43	3	3	-13
Alta Verapaz	8,578	593	120	605	106	514	121	532	127	743	155	750	138	2	3	1
Chimaltenango	8,236	653	83	670	78	576	157	636	114	116	88	120	69	3	10	3
Baja Verapaz	7,236	741	84	725	96	753	97	750	95	414	275	402	272	-2	0	-3
San Marcos	5,992	499	222	574	192	315	248	363	242	171	207	171	207	15	15	0
El Progreso	5,366	565	143	540	135	551	150	533	97	196	231	232	276	-4	-3	19
Zacapa	4,178	633	167	640	157	630	124	655	114	452	301	448	295	1	4	-1
Izabal	3,273	621	128	568	149	614	108	615	106	742	73	735	83	-9	0	-1
Totonicapán	3,255	583	272	678	180	322	313	394	297	47	84	49	83	16	22	4
Sololá	2,902	588	170	669	124	353	255	432	237	100	129	99	125	14	23	-1
Quetzaltenango	2,502	552	174	544	235	321	223	334	182	167	174	156	177	-1	4	-6
Sacatepéquez	1,430	680	38	702	50	593	129	658	98	102	39	116	30	3	11	14
Escuintla	907	580	151	514	146	582	116	536	162	176	61	151	73	-11	-8	-14
Total	208,084												average	1	6	-2
													50% area	-2	5	-8
													> 10% loss	10	0	20
