# Supplementary material for: Assessing high-impact spots of climate change: spatial yield simulations with Decision Support System for Agrotechnology Transfer (DSSAT) model
Source: Mitig Adapt Strateg Glob Chang. 2016 Feb 6;22(5):743–60. doi: 10.1007/s11027-015-9696-2 (PMC6054003; doi:10.1007/s11027-015-9696-2)

Mitigation and Adaptation Strategies for Global Change:

Assessing high impact spots of climate change: spatial yield simulations with Decision Support System for Agrotechnology Transfer (DSSAT) model.

Anton Eitzinger, Peter Läderach, Beatriz Rodriguez, Myles Fisher, Stephen Beebe, Kai Sonder, Axel Schmidt

Anton Eitzinger, CIAT International Center for Tropical Agriculture, Cali, Colombia; a.eitzinger@cgiar.org

---

Online Resource 2:

We present maps for drybeans Impact Hot Spots (IHS) for different planting seasons in Central America.

Impact Hot Spots (HIS) as positive (green) and negative (red) outliers from yield change of drybeans in Central America, *postrera* season. Hatched areas are the main bean growing areas; white points are the 15 selected bean production sites.

First planting season Primera (May to July)

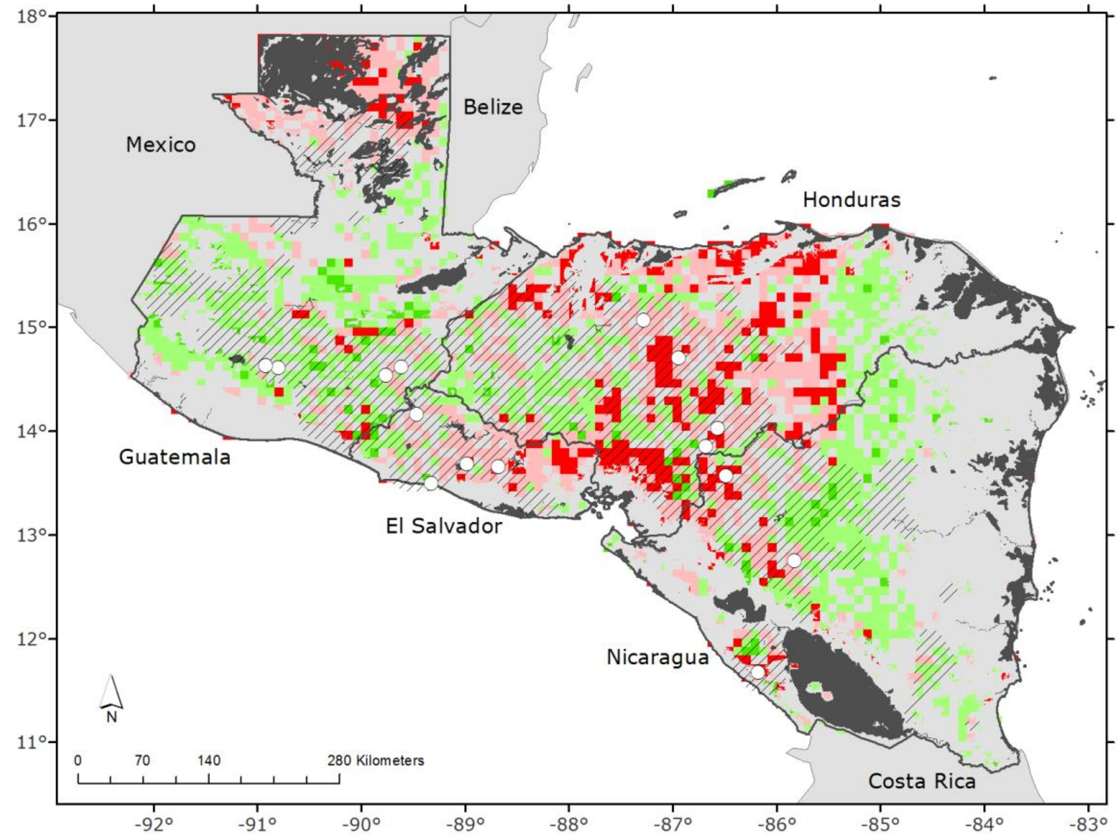

Second planting season Postrera (September to November)

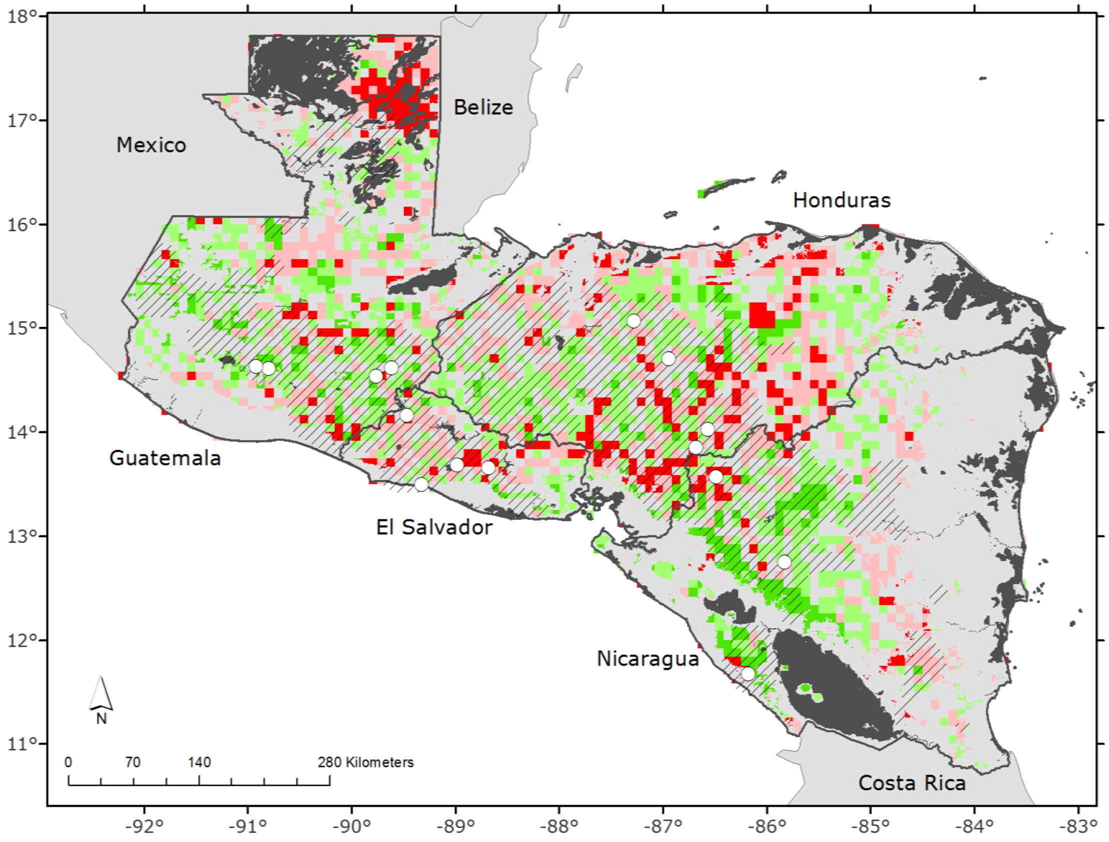

Third planting season Apante (December to February)

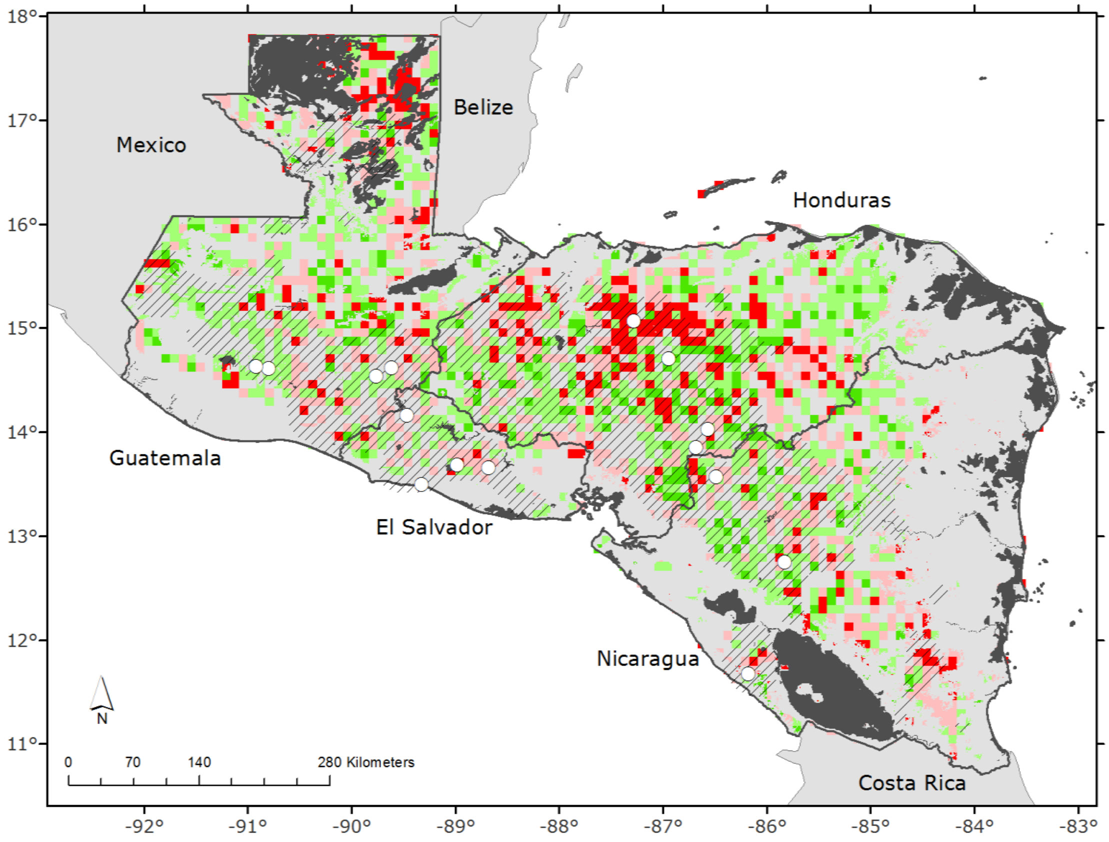

Supplement: Supplementary file 2 — Maps of drybeans impact hot spots HIS for three planting seasons in four Central American countries. (PDF 943 kb) [file 11027_2015_9696_MOESM2_ESM.pdf]
